# History of Childhood Abuse, Sensation Seeking, and Intimate Partner Violence under/Not under the Influence of a Substance: A Cross-Sectional Study in Russia

**DOI:** 10.1371/journal.pone.0068027

**Published:** 2013-07-02

**Authors:** Weihai Zhan, Alla V. Shaboltas, Roman V. Skochilov, Tatiana V. Krasnoselskikh, Nadia Abdala

**Affiliations:** 1 Center for Interdisciplinary Research on AIDS, Yale School of Public Health, New Haven, Connecticut, United States of America; 2 Department of Psychology, Saint Petersburg State University, Saint Petersburg, Russian Federation; 3 Department of Dermatology and Venereal Diseases, Saint Petersburg Pavlov State Medical University, Saint Petersburg, Russian Federation; Indiana University and Moi University, United States of America

## Abstract

**Objectives:**

To examine correlates of perpetration and victimization of intimate partner violence (IPV) under and not under the influence of a substance, we conducted a study among women in Russia.

**Methods:**

In 2011, a cross-sectional survey was conducted among patients receiving services at a clinic for sexually transmitted infections in St. Petersburg, Russia. Multinomial logistic regression was used for analysis.

**Results:**

Of 299 women, 104 (34.8%) and 113 (37.8%) reported a history of IPV perpetration and victimization, respectively. Nearly half (47.1%) of perpetrators and 61.1% of victims reported that the latest IPV event (perpetration and victimization, respectively) was experienced under the influence of a substance. Factors independently associated with IPV victimization under the influence of a substance were alcohol misuse and a higher number of lifetime sex partners, whereas only experience of childhood abuse (emotional and physical abuse) was independently associated with IPV victimization that did not occur under the influence of a substance. Childhood physical abuse, lower age of first sex, sensation seeking, and alcohol misuse were independently associated with IPV perpetration under the influence of a substance, while only childhood abuse (emotional and physical abuse) was independently associated with IPV perpetration that did not occur under the influence of a substance.

**Conclusions:**

IPV under and not under the influence of a substance had different correlates (e.g., alcohol misuse and sensation seeking). Despite the strong association between substance use and IPV, experience of childhood abuse is an important predictor of IPV perpetration and victimization in Russia, above and beyond substance use.

## Introduction

Intimate partner violence (IPV) has been a significant public concern worldwide due to its high prevalence and wide range of adverse health consequences such as injury, chronic pain, sexually transmitted infections (STIs) and mental health problems [1]. Findings from the National Violence Against Women Survey revealed that 25% of U.S. women and 8% of U.S. men reported a history of being raped and/or physically assaulted by a spouse or partner at some time in their lives [2]. In Russia, results from the limited studies conducted among several different samples all demonstrate a high lifetime prevalence (from 23% perpetration and 26% victimization among STI patients to 46% perpetration among HIV-infected substance-using men), which calls for more research to examine factors related to IPV in this country [3], [4], [5], [6].

Although a variety of studies revealed that a number of factors are associated with IPV [7], substance (alcohol and drug) use is the key factor that is most consistently and strongly linked to IPV [8], [9]. Substance use is associated with IPV perpetration and victimization indirectly through facilitating the occurrence of conflicts within relationships (e.g., exacerbating financial difficulties, childcare problems or other family stressors) [10] or directly through psychopharmacological effects on cognitive functioning (i.e., reducing the ability for negotiating conflicts) [11]. Considerable empirical evidence shows that substance use often precedes or accompanies acts of IPV [12], [13]. For example, a U.S. national study found that 30% to 40% of the male perpetrators and 27% to 34% of the female perpetrators were drinking at the time of the IPV event [14].

Experience of childhood abuse, particularly physical abuse, is another key factor that has been found to be consistently associated with IPV perpetration in different studies, including a cross-sectional study using data collected from a large representative U.S. national sample of couples (n = 1,635) and a 20-year longitudinal study [15], [16], [17], [18]. It has been proposed: (1) that people may acquire a propensity to perpetrate violence through the experience of childhood abuse, and (2) that abused children are less likely to develop the ability of self-control and skills of negotiation [19]. Although much attention has been paid to the relationship between childhood abuse and IPV perpetration, less research has investigated whether childhood abuse increases risk of IPV victimization [15], [20], [21]. Social learning theory indicates that experiencing abuse as a child may cause IPV victimization through the development of tolerance of violence within the family [22].

Sensation seeking is another factor associated with IPV. This association is less consistent in the literature partly because IPV is a complex phenomenon which may not be fully explained by a single factor or simple model [7], [23], [24]. For example, sensation seeking was associated with IPV perpetration among couples in one study [25], but not in another study conducted among male undergraduates [26]. In a third study, women with higher sensation-seeking scores reported a higher prevalence of IPV victimization whereas no significant difference of IPV victimization prevalence between high and low levels of sensation-seeking scores was observed among men [27]. Because sensation seeking is a personality trait commonly associated with problem behaviors such as substance use and sexual risk behaviors [28], [29], the investigation of the association between sensation seeking and IPV may provide useful information for IPV prevention studies.

Substance use, particularly alcohol and drug use, is an important public health problem in Russia, and has been associated with several negative outcomes including IPV and HIV risk behaviors [6], [30]. Our previous study has shown that substance use plays a key role in IPV in Russia [31]. However, to this date no study has examined factors associated with IPV under and not under the influence of a substance in Russia.

This study used multinomial logistic regression to simultaneously investigate three levels of outcome: IPV under the influence of a substance, IPV not under the influence of a substance, and no IPV. The primary purpose of the present study was to examine correlates of perpetration and victimization of IPV under and not under the influence of a substance (each compared to no IPV) among women receiving health services in St. Petersburg, Russia. We hypothesized that: (1) IPV under and not under the influence of a substance has different correlates; (2) experience of childhood abuse is associated with IPV perpetration and victimization with and without the influence of a substance; and (3) sensation seeking is associated with IPV perpetration independently of whether it occurs under the influence of a substance.

## Methods

### Participants and Measures

Consecutive adult patients (aged 18 years and older) who required STI services in a dermatovenereology dispensary (STI out-patient clinic) in St. Petersburg, Russia were invited to participate in a cross-sectional study which aimed to examine factors associated with female reproductive health from May 2011 to November 2011. A total of 502 patients agreed to participate in and completed a computer-assisted interviewer-administered questionnaire. Written informed consent was obtained from all participants and the study was approved by the institutional review boards of the Biomedical Center in St. Petersburg, Russia and Yale University, CT, USA. Of the 300 women recruited into the study, 299 answered the questions about intimate partner violence and therefore comprised the final analytic sample. The decision to include only women in the present analyses was made to reduce the complexity of research questions because women and men had different recruitment criteria. Eligibility for women’s participation included: (1) age between 18 and 50; (2) sexually active during the past 6 month; (3) not trying to get pregnant; and (4) biologically able to have children.

A questionnaire was used to collect information including demographic and health characteristics, alcohol and illicit drug use, sexual behaviors, sensation seeking and experience of violence including history of childhood abuse and IPV. The questionnaire was constructed in English, translated to Russian, and then translated back to English to ensure the integrity of the syntax and meaning. Demographic information included age at survey, marital status, level of education, employment status and income level.

*Alcohol and illicit drug use:* Alcohol consumption was assessed using the Alcohol Use Disorders Identification Test (AUDIT) which includes 10 questions with a total score that ranges from 0 to 40 [32]. A score of 8 or higher is usually indicative of alcohol misuse [32]. The Cronbach’s α for the AUDIT was 0.87 in the current sample. Participants were also asked whether they had ever used illicit drugs.

*Sexual behaviors:* Participants were asked the age of their first sexual experience and the number of sexual partners in their lifetime. Considering that some participants had a much higher number of sexual partners, this variable was dichotomized according to the median value.

*Sensation seeking*: The Brief Sensation Seeking Scale (BSSS-8) developed by Hoyle et al. was used to measure sensation seeking [33]. The BSSS-8 contains 8 Likert-type items, including “I would like to explore strange places,” “I would like to take off on a trip with no preplanned routes or timetables,” “I get restless when I spend too much time at home,” “I prefer friends who are exciting and unpredictable,” “I like to do frightening things,” “I would like to try bungee jumping,” “I like wild parties,” and “I like new and exciting experiences even if I have to break the rules.” All items ranged from 1 =  “strongly disagree” to 5 =  “strongly agree” and the BSSS-8 was obtained by averaging responses to all items. The scale was internally consistent in the current sample (Cronbach’s α = 0.77).

*History of childhood abuse:* We assessed experiences of childhood abuse from two aspects, emotional abuse and physical abuse, respectively, based on two questions: “How often did your parent or caretaker insult, swear or threaten you” and “How often did your parent or caretaker push, grab, pinch or beat you” during their first 18 years. Although we asked a question related to childhood sexual abuse, “How often did your parent or caretaker touch you in a sexual way or had you touch him/her in a sexual way,” only one woman had such an experience. Thus, childhood sexual abuse was not included in the present analysis. These questions were adapted from the Conflict Tactics Scale (CTS) and the response categories were *never*, *rarely*, *frequently* and *way too frequently* [34]. The presence or absence of each type of childhood abuse was dichotomized as No–never vs. Yes–any of the three positive responses.

*Perpetration and victimization of IPV:* IPV perpetration was defined as having ever (a) insulted, sworn at, or threatened a sexual partner; (b) pushed, grabbed, slapped, punched, beaten up, or choked a sexual partner; or (c) physically forced a sexual partner to have sex or do something sexually that he or she did not want to do. IPV victimization was defined as having ever been the target of the aforementioned actions by a sexual partner. These partner violence items were adapted from the CTS and have been successfully used in Russia to measure intimate partner violence [31], [34]. Those who answered “yes” to any one of the three items were further asked, “The last time this happened, were you or your partner high on alcohol or drugs?” An answer of “yes” was classified as IPV under the influence of a substance.

### Data Analysis

Chi-square tests and ANOVA (analysis of variance) F-tests were used to examine group differences by last IPV status (e.g., last perpetration under and not under the influence of a substance and no perpetration) for categorical and continuous variables, respectively. Multinomial logistic regression was used to determine significant correlates of IPV under and not under the influence of a substance. None of the demographic characteristics were significantly associated with either IPV perpetration or victimization in the multinomial logistic regression; therefore they were not included in the final models. The significance level was defined as *p*<.05 and the data were analyzed using SAS software version 9.1 (SAS Institute Inc., 2003).

## Results

### Demographic Characteristics, Experience of Childhood Abuse and IPV Perpetration and Victimization

The mean age of the participants was 28 years (standard deviation = 7.8), ranging from 18 to 50 years. Nearly 50% of the participants were married, 42% were never married and the rest were divorced, widowed or separated. About 49% of the participants had completed university or higher education. The majority (57%) of the participants were full-time employed and 43% had monthly incomes below 15,000 rubles (about 500 U.S. dollars). None of these demographic characteristics differed by status of last IPV perpetration (i.e., under and not under the influence of a substance and no perpetration). When these demographic characteristics were compared by status of last IPV victimization (i.e., under and not under the influence of a substance and no victimization), however, age of the participants was significantly different (mean age 26.7, 30.9 and 27.6 years for participants under and not under influence of substance and no victimization, respectively, *p* = 0.01). Other characteristics were not significantly different across status of last IPV victimization.

The prevalence of childhood abuse was 63.5% (190 of 299). Among 190 women who had experience of childhood abuse, 118 (62.1%) only had experience of childhood emotional abuse and 72 (37.9%) had childhood physical abuse experience with 70 of 72 reporting both experiences.

Both IPV perpetration and victimization were common. Of the women in the study, one did not answer IPV questions; 104 (34.8%) reported a history of IPV perpetration and 113 (37.8%) reported a history of IPV victimization, with 75 (25.1%) reporting both perpetration and victimization experiences in their lives. More specifically, the percentages of women who experienced verbal, physical and sexual IPV were 32.1, 18.1 and 11.0%, respectively; the percentages of women who perpetrated verbal, physical and sexual IPV were 28.4, 21.4 and 2.7%, respectively. Of 104 women who had ever perpetrated IPV, 49 (47.1%) reported last perpetration under the influence of a substance. Among 113 women who had ever been a victim of IPV, 69 (60.6%) reported last victimization under the influence of a substance.

### Correlates of IPV Perpetration under and not under the Influence of a Substance

Experience of childhood abuse, sensation seeking, illicit drug use, alcohol misuse, age at first sex and number of lifetime sex partners significantly differed by status of last IPV perpetration, with *p*-values ranging from <.0001 to 0.001 (Table 1). Multinomial logistic regression shows that experience of childhood physical abuse, lower age of first sex, sensation seeking, and alcohol misuse were significantly associated with last perpetration of IPV under the influence of a substance, while only experience of childhood abuse (emotional and physical abuse) was significantly associated with last perpetration of IPV not under the influence of a substance (Table 2).

**Table 1:** Bivariate analyses of intimate partner violence (IPV) perpetration by childhood abuse, sensation seeking, substance use, and sexual factors (N = 299).^a^

Variables	Perpetration under influenceof a substance (n = 49)	Perpetration not under influenceof a substance (n = 55)	No perpetration(n = 195)	*p*-value^b^
Childhood abuse				0.001
Any physical abuse	34.7%	36.4%	17.9%	
Emotional abuse only	36.7%	45.4%	38.5%	
No abuse	28.6%	18.2%	43.6%	
Sensation seeking	3.8 (0.8)	3.3 (1.0)	3.0 (0.8)	<.0001
Illicit drug use	55.1%	20.0%	19.5%	<.0001
Alcohol misuse	55.1%	10.9%	9.2%	<.0001
Age at first sex (years)	16.1 (1.4)	16.9 (1.6)	17.5 (1.9)	<.0001
≥6 lifetime sex partners	63.3%	49.1%	33.3%	0.0003

a The numbers in the table are proportions for variables except for age at first sex and sensation seeking in which mean and standard deviation (SD) were presented.

b *p*-value was obtained using Chi-square tests for categorical variables and ANOVA (analysis of variance) F-tests for continuous variables (i.e., age at first sex and sensation seeking).

**Table 2:** Multinomial logistic regression: correlates of perpetration of intimate partner violence (IPV) under and not under the influence of a substance (N = 299).^a^

Variables	Perpetration under influenceof a substance b	Perpetration not under influenceof a substance^b^
Childhood abuse		
No abuse	1.00	1.00
Emotional abuse only	1.43 (0.58–3.55)	2.88 (1.28–6.48)
Any physical abuse	2.83 (1.05–7.59)	4.74 (1.99–11.31)
Sensation seeking	2.13 (1.31–3.47)	1.31 (0.90–1.92)
Illicit drug use	2.16 (0.97–4.81)	0.69 (0.30–1.58)
Alcohol misuse	4.71 (2.04–10.86)	0.75 (0.26–2.14)
Age at first sex (years)	0.73 (0.55–0.95)	0.85 (0.69–1.03)
≥6 lifetime sex partners	1.68 (0.77–3.70)	1.72 (0.88–3.35)

a Odds ratio and 95% confidence interval were presented in the table. None of the demographic characteristics were significant in the multinomial logistic regression and thus were not included in the final model.

b The reference category is no IPV.

### Correlates of IPV Victimization under and not under the Influence of a Substance

Experience of childhood abuse, sensation seeking, illicit drug use, alcohol misuse, age at first sex and number of lifetime sex partners significantly differed by status of last IPV victimization, with *p*-values ranging from <.0001 to 0.01 (Table 3). Multinomial logistic regression shows that alcohol misuse and a higher number of lifetime sex partners were significantly associated with last victimization of IPV under the influence of a substance, while only experience of childhood abuse (emotional and physical abuse) was significantly associated with last victimization of IPV not under the influence of a substance (Table 4).

**Table 3:** Bivariate analyses of victimization by intimate partner violence (IPV) by childhood abuse, sensation seeking, substance use, and sexual factors (N = 299).^a^

Variables	Victimization under influenceof a substance (n = 69)	Victimization not under influenceof a substance (n = 44)	No victimization(n = 186)		*p*-value^b^
Childhood abuse					0.01
Any physical abuse	31.9%	34.1%	18.8%		
Emotional abuse only	36.2%	47.7%	38.7%		
No abuse	31.9%	18.2%	42.5%		
Sensation seeking	3.6 (0.9)	3.1 (0.9)	3.1 (0.8)		0.0003
Illicit drug use	42.0%	27.3%	18.8%		0.0007
Alcohol misuse	43.5%	13.6%	8.1%		<.0001
Age of first sex (years)	16.4 (1.9)	17.1 (1.6)	17.4 (1.8)		0.0005
≥6 lifetime sex partners	60.9%	50.0%	31.7%	<.0001	

a The numbers in the table are proportions for variables except for age at first sex and sensation seeking in which mean and standard deviation (SD) were presented.

b *p*-value was obtained using Chi-square tests for categorical variables and ANOVA (analysis of variance) F-tests for continuous variables (i.e., age at first sex and sensation seeking).

**Table 4:** Multinomial logistic regression: correlates of victimization of intimate partner violence (IPV) under and not under the influence of a substance (N = 299).^a^

Variables	Victimization under the influence of a substance^b^	Victimization not under the influence of a substance^b^
Childhood abuse		
No abuse	1.00	1.00
Emotional abuse only	1.20 (0.58–2.50)	3.01 (1.24–7.29)
Any physical abuse	1.89 (0.85–4.21)	4.04 (1.55–10.54)
Sensation seeking	1.34 (0.92–1.97)	0.90 (0.59–1.36)
Illicit drug use	1.48 (0.73–3.00)	1.28 (0.55–2.94)
Alcohol misuse	4.81 (2.19–10.57)	1.39 (0.47–4.14)
Age of first sex (years)	0.86 (0.70–1.06)	0.95 (0.77–1.16)
≥6 lifetime sex partners	2.13 (1.11–4.07)	1.89 (0.92–3.90)

a Odds ratio and 95% confidence interval were presented in the table. None of the demographic characteristics were significant in the multinomial logistic regression and thus were not included in the final model.

b The reference category is no IPV.

To exclude the possibility that the null association between experience of childhood abuse and IPV victimization under the influence of a substance was caused by the mediation effect of alcohol misuse, we excluded alcohol misuse from the final model. In this case, the null association between childhood abuse and IPV victimization under the influence of a substance remained.

## Discussion

Although the strong effect of substance use, particularly alcohol misuse, on IPV observed in the present study is consistent with former research [8], [9], [10], [31], [35], no previous studies have simultaneously examined correlates of IPV under and not under the influence of a substance in Russia. In line with our expectations, IPV under and not under the influence of a substance had different correlates. We also found that experience of childhood abuse was a shared risk factor for IPV under and not under the influence of a substance, above and beyond substance use. Sensation seeking, however, was only significantly associated with IPV perpetration under the influence of a substance, but not without the influence of a substance.

As expected, our findings showed a strong association between alcohol misuse and IPV under the influence of a substance. However, the result showed no association between alcohol misuse and IPV not under the influence of a substance. This suggests that IPV not under the influence of a substance is equally likely to be reported by women who misused or did not misuse alcohol. This may have implications for IPV prevention strategies. For example, interventions to prevent IPV may be more effective by reducing alcohol misuse among perpetrators or victims with a history of IPV under the influence of a substance, rather than among female perpetrators/victims who misuse alcohol but do not commit IPV under the influence of a substance. On the other hand, interventions for females who misuse alcohol and are IPV perpetrators/victims not under the influence of a substance may be more effective when emphasizing other factors, such as adverse effects of experience of childhood abuse, which is also indicated by our results.

The majority of studies investigating the relationship between childhood abuse and IPV have focused on childhood physical and/or sexual abuse [19], [20], [21], [36], though childhood emotional abuse was occasionally examined [37], [38]. In our study, only one woman experienced childhood sexual abuse and thus this type of childhood abuse was not included in our analyses. Our results suggest childhood abuse predicted both IPV perpetration and victimization above and beyond the contribution of substance use. Additionally, experience of childhood abuse may play a more important role in IPV not under the influence of a substance than IPV under the influence of a substance. Among a series of potential correlates examined in the present study, including socio-demographic characteristics, substance use, childhood abuse, sensation seeking and sexual history, experience of childhood abuse was the only significant correlate for IPV perpetration and victimization not under the influence of a substance. This was not the case for IPV perpetrators/victims with the influence of substance. Our results provide new evidence supporting that even experience of childhood emotional abuse only (no physical abuse) may also be at risk for IPV. One study conducted among a large, ethnically diverse, college student sample demonstrated that childhood emotional abuse was an even stronger predictor of IPV than childhood physical abuse [38]. Taken together, our data suggest that screening for a history of childhood abuse including emotional abuse, is needed as part of an assessment for IPV.

Our study provides strong evidence of an association between sensation seeking and IPV perpetration even after controlling for the effects of substance use and childhood abuse on IPV. The result is similar to a previous study conducted among a large sample of young adults who participated in the Wave III of the National Longitudinal Study of Adolescent Health (NLSAH), in which couples with experience of IPV had higher sensation seeking scores than those without IPV experience [25]. However, because important confounding factors in the NLSAH such as substance use were not controlled for, their results may have been biased. Thus, the results of our study provide more direct and stronger evidence for the association between sensation seeking and IPV perpetration.

As with most studies, our study is not without limitations. First and foremost, our data may be subject to recall bias. The measurement of experience of childhood abuse and last IPV under and not under the influence of a substance was based upon self-reporting. It is possible that participants may not accurately recall their exposure to violence in their childhood and exposure to substance if the last IPV occurred many years ago. The misclassification resulting from recall bias is likely to attenuate the observed association. Second, the measurement of IPV may also be subject to social desirability bias. As a result, IPV may be underreported. Furthermore, some participants may use substances as an excuse for their IPV acts even in a situation where no substance was actually involved in their last IPV episode. However this possibility should be low since the proportion of IPV under the influence of a substance in the present study is consistent with results from other studies [12], [14]. Third, the way we used to define IPV under and not under the influence of a substance does not allow us to discern who (the participant or her partner) used the substance and which substance (alcohol or illicit drug) was used in their last IPV. Further discrimination which requires a larger sample size may provide more insights into the nature of IPV. A larger sample size can also allow us to directly compare correlates of IPV under the influence of a substance and correlates of IPV not under the influence of a substance. Fourth, although we measured substance use during the last IPV event, data regarding the frequency of IPV under the influence of a substance had not been collected. Therefore, the most recent incident may have a disproportionate impact on the results of the analysis. Other important limitations include the cross-sectional design and limited generalizability of our results. For example, the cross-sectional design does not allow us to make an inference on the direction for the association between more sexual partners and IPV victimization under the influence of a substance. Also the non-random sample used in the present study may not allow any generalization of our results. Moreover, particular caution should be taken with respect to the fact that the sample was enrolled in an STI clinic, and it is unknown whether a similar finding would emerge from the general population or from individuals recruited in other types of clinics.

In conclusion, IPV under and not under the influence of a substance had different correlates (e.g., alcohol misuse and sensation seeking) and experience of childhood abuse can be an important predictor of IPV perpetration and victimization, above and beyond substance use. The findings also support the growing body of work suggesting that, in addition to childhood physical abuse, childhood emotional abuse only can be a risk factor of IPV which warrants more research and clinical attention.
